# Malaria prevalence and performance of diagnostic tests among patients hospitalized with acute undifferentiated fever in Zanzibar

**DOI:** 10.1186/s12936-022-04067-z

**Published:** 2022-02-19

**Authors:** Annette Onken, Christel Gill Haanshuus, Mohammed Khamis Miraji, Msafiri Marijani, Kibwana Omar Kibwana, Khamis Ali Abeid, Kristine Mørch, Marianne Reimers, Nina Langeland, Fredrik Müller, Pål A. Jenum, Bjørn Blomberg

**Affiliations:** 1 Department of Clinical Science, University of Bergen, Bergen, Norway; 2 Norwegian National Advisory Unit on Tropical Infectious Diseases, Department of Medicine, Haukeland University Hospital, Bergen, Norway; 3 Department of Microbiology, Vestre Viken Hospital Trust, Postbox 800, 3004 Drammen, Norway; 4 Department of Internal Medicine, Mnazi Mmoja Hospital, Zanzibar, Tanzania; 5 Pathology Laboratory Department, Mnazi Mmoja Hospital, Zanzibar, Tanzania; 6 Department of Paediatrics, Mnazi Mmoja Hospital, Zanzibar, Tanzania; 7 Department of Medicine, Haukeland University Hospital, Bergen, Norway; 8 Department of International Collaboration, Haukeland University Hospital, Bergen, Norway; 9 Department of Microbiology, Oslo University Hospital, Oslo, Norway; 10 Institute of Clinical Medicine, University of Oslo, Oslo, Norway

**Keywords:** Malaria, Prevalence, Surveillance, Fever, Microscopy, Point-of-care diagnostic tests, Polymerase chain reaction, Zanzibar, Tanzania, Eastern Africa

## Abstract

**Background:**

Control efforts in Zanzibar reduced the burden of malaria substantially from 2000 to 2015, but re-emergence of falciparum malaria has been observed lately. This study evaluated the prevalence of malaria and performance of routine diagnostic tests among hospitalized fever patients in a 1.5 years period in 2015 and 2016.

**Methods:**

From March 2015 to October 2016, paediatric and adult patients hospitalized with acute undifferentiated fever at Mnazi Mmoja Hospital, Zanzibar were included. The malaria prevalence, and performance of rapid diagnostic test (RDT) and microscopy, were assessed using polymerase chain reaction (PCR) as gold standard.

**Results:**

The malaria prevalence was 9% (63/731). Children under 5 years old had lower malaria prevalence (5%, 14/260) than older children (15%, 20/131, p = 0.001) and persons aged 16 to 30 years (13%, 15/119, p = 0.02), but not different from persons over 30 years old (6%, 14/217, p = 0.7). All cases had *Plasmodium falciparum* infection, except for one case of *Plasmodium ovale.* Ten malaria patients had no history of visiting mainland Tanzania. The RDT had a sensitivity of 64% (36/56) and a specificity of 98% (561/575), and microscopy had a sensitivity of 50% (18/36) and  a specificity of 99% (251/254), compared to PCR. The malaria parasitaemia was lower in patients with false negative results on RDT (median 7 × 10^3^ copies/µL, interquartile range [IQR] 2 × 10^3^ – 8 × 10^4^, p = 0.002) and microscopy (median 9 × 10^3^ copies/µL, IQR 8 × 10^2^ – 7 × 10^4^, p = 0.006) compared to those with true positive RDT (median 2 × 10^5^ copies/µL, IQR 3 × 10^4^ – 5 × 10^5^) and microscopy (median 2 × 10^5^ copies/µL, IQR 6 × 10^4^ – 5 × 10^5^).

**Conclusions:**

The study emphasizes that malaria was a frequent cause of febrile illness in hospitalized patients in Zanzibar in the years 2015-2016, particularly among school age children and young adults. We found evidence of autochthonous malaria transmission in Zanzibar. Compared to PCR, both RDT and microscopy had low sensitivity, and false negative results were associated with low parasitaemia. While low parasitaemia identified only by PCR in a semi-immune individual could be coincidental and without clinical relevance, clinicians should be aware of the risk of false negative results on routine tests.

## Background

Successful control efforts reduced the burden of malaria in Zanzibar substantially from 2000 to 2015 [1]. However, this progress has halted in recent years. According to the World Health Organization (WHO), sub-Saharan Africa suffered 384,000 estimated malaria deaths in 2020, equalling 94% of the global malaria death toll [2, 3]. In the Zanzibar archipelago, a comprehensive control and elimination programme was implemented in 2001, introducing artemisinin-based combination therapy, intermittent treatment in pregnancy, nationwide distribution of long-lasting insecticide-treated bed nets, indoor residual spraying, active case detection among contacts and larvicidal treatment of mosquito breeding sites [4]. The interventions reduced malaria-prevalence by 96% from 2002 to 2015 [5], malaria in-patient cases by 78% from 1999 to 2008 [6], and cut reported deaths to negligible. However, since 2016 the number of reported cases in Zanzibar has increased [2], and in 2020, the Zanzibar Ministry of Health intensified control measures after a surge in malaria cases during a prolonged rainy season.

Commercially available malaria rapid diagnostic tests (RDTs) differ widely in sensitivity and specificity [7], and accurate microscopy depends on high quality technical equipment and experience [8]. While the sensitivity of polymerase chain reaction (PCR) also varies between assays, PCR has generally high sensitivity and detect parasitaemia lower than 1 parasite/µL, while the detection limits for microscopy and sensitive RDTs are around 50–200 p/µL and 100 p/µL, respectively [9].

The main objective of this study was to evaluate the prevalence of malaria identified by PCR, and the performance of the routine tests RDT and microscopy, in febrile patients admitted to Mnazi Mmoja Hospital (MMH), Zanzibar.

## Methods

### Patient material

From 17th March 2015 to 4th October 2016, we consecutively enrolled patients with acute undifferentiated febrile illness admitted to the Department of Internal Medicine and the Department of Paediatrics at MMH. With 544 beds, this hospital in Zanzibar city is the referral hospital for the 1.3 million population of the Zanzibar Archipelago [10]. Inclusion criteria were fever (≥ 38.3 °C in adults, ≥ 38.5 °C in children) or hypothermia (< 36.0 °C), tachypnoea > 20/min, tachycardia > 90/min on admission, or attending clinicians’ diagnosis of severe acute infection. Neonates under 15 days of age were excluded. Demographic and clinical information was obtained using a standardized case-report form.

Blood for on-site RDT and microscopy, and blood in EDTA tubes was obtained, the latter stored at − 20 °C and shipped on dry ice to Norway for malaria-PCR to be done later. Malaria microscopy was performed if requested by attending clinician, while PCR and RDT was performed on all patients for the sake of the study.

PCR was defined as gold standard for assessment of prevalence and for evaluation of performance of routine diagnostic tests.

### Microscopy and rapid diagnostic test

For microscopy, a 10% Giemsa solution was used to stain both thick and thin blood films, in accordance with hospital procedures. The RDT First Response Malaria Ag pLDH/HRP2 Combo Card Test (Premier Medical Corporation Ltd., India) was used until 20.08.2016. At this time, for the remaining 6 weeks, it was replaced by CareStartTM Malaria HRP2/pLDH (Pf/PAN) Combo (Access Bio, Inc., Somerset, NJ, USA) due to stock-out. 96% (685/714) of the patients were tested with the first RDT.

### PCR methods

DNA was extracted from 500 µL whole blood using MagNA Pure 96 DNA and Viral NA Large Volume Kit (Roche Diagnostics GmbH, Mannheim, Germany). Presence of *Plasmodium* DNA was assessed applying a genus-specific PCR, targeting *cytochrome b (cytb)* on the mitochondrial genome, and quantitative analysis (q-PCR) was performed using a customized plasmid, as previously described [11]. Parasitaemia by PCR was given in copies/µL blood as unit of measurement. The mitochondrial *cytb* target exists in about 20–160 copies depending on the different development stages. One mitochondrion harbours about 20 copies of the *Plasmodium* genome. It is reported that early ring stage parasites have one mitochondrion, while mature gametocytes have up to eight fold higher quantity of the mitochondrial genome (about 80–160 copies) [12, 13]. Due to unknown variation of development stages in a sample, the unit copies/µL blood cannot be converted into the unit parasites/µL. For quality assurance, results with cycle threshold values ≥30 (low amplification) were re-analysed in triplicates, and in case of discordant results between PCR, RDT or routine microscopy, samples were retested from DNA extraction, using QIAamp DNA Blood Mini Kit (Qiagen, Hilden, Germany) according to the manufacturer’s instructions. Genus-specific PCR positive samples were further analysed by species-specific real-time PCR assays targeting the 18 S gene of *Plasmodium falciparum*, *Plasmodium vivax, Plasmodium ovale* and *Plasmodium malariae*. Previously published primers [14, 15] were applied in separate master mixes with the following concentrations: 200 nM for *P. falciparum*, 100 nM for *P. vivax*, 300 nM for *P. ovale*, and 200 nM for *P. malariae*. The species-specific amplifications were performed using the following cycling parameters: Step 1, 50 °C for 2 min; step 2, 95 °C for 10 min; step 3, denaturation at 95 °C for 15 s and step 4, annealing at 60 °C for 1 min; steps 3–4 repeated 40 times. All reaction mixtures, both genus-/ and species-specific, contained 2 µL template (DNA), and 12.5 µL SYBR Select Master Mix (Applied Biosystems, Carlsbad, CA, USA), at a total volume of 25 µL. To identify species in samples not detected by species-specific PCR, relevant genus-specific positive PCR-products were sequenced in one direction applying primer PgMt19 F3, sequences run by BLAST, and specific polymorphisms confirmed, as previously described [14].

### Statistics

Dichotomous variables were assessed by Chi-square test, and by binomial logistic regression for factors with multiple levels (i.e. age groups). Continuous variables such as age and level of parasitaemia, were assessed by pairwise Wilcoxon rank-sum test for two groups and by Kruskal Wallis test for multiple groups. Analyses were performed in R version 4.1.2, Rstudio version 2021.09.1 (R Core Team, Vienna, Austria) [16]. 

## Results

**Fig. 1:**
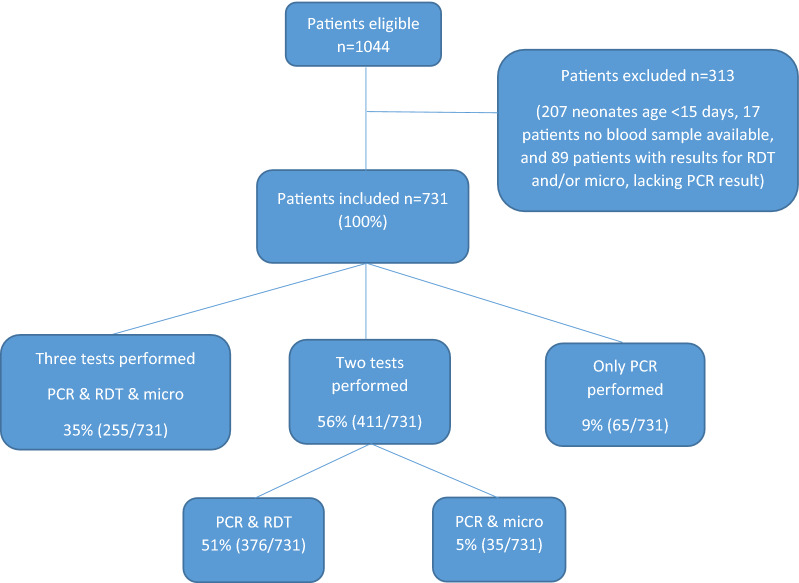
Patients included and analyses performed

Among 1044 patients fulfilling the inclusion criteria, we excluded 207 neonates < 15 days, 17 patients from whom we could not obtain blood for testing and 89 patients lacking a result for PCR, resulting in a study population of 731 (Fig. 1). 58% (421/731) were males. Median age was 13 years, range 16 days–95 years. 50% were admitted to the Department of Paediatrics (n = 362, age range 16 days–14 years) and 50% to the Department of Internal Medicine (n = 369, age range 13–95 years).

**Fig. 2:**
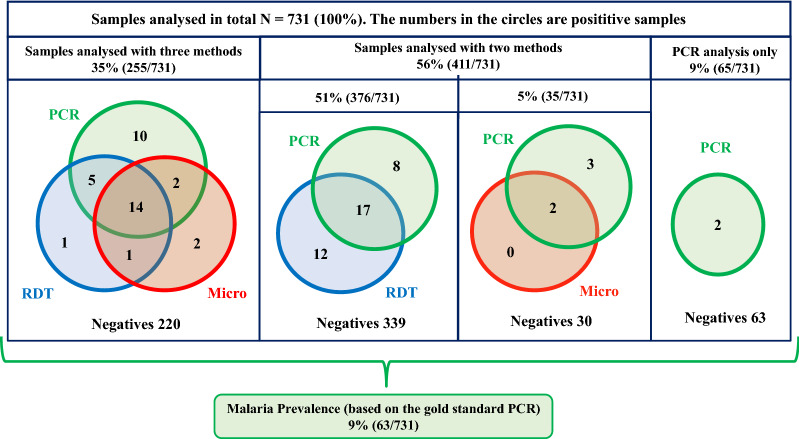
An overview and results from the analyses performed by PCR, RDT and routine microscopy. The numbers in the circles refer to malaria positive results performed by each method. The numbers of malaria negatives are given in the bottom of the squares. Except for the malaria prevalence, all numbers are given for the performance of each method independently of false positives/negatives by the gold standard method PCR

Malaria prevalence defined by positive PCR was 9% (63/731) (Fig. 2). The median parasitaemia was 5 × 10^4^ copies/µL blood (interquartile range [IQR] 4 × 10^3^ to 4 × 10^5^). All positive cases had *P. falciparum* infection, except for one, who had *P. ovale* identified by sequencing (parasitaemia 1.1 × 10^5^ copies/µL blood, negative RDT, positive microscopy). Malaria RDT was performed in 631 patients, of whom 8% (50/631) had positive test results. Compared to PCR, the RDT had a sensitivity of 64% (36/56), a specificity of 98% (561/575), a positive predictive value (PPV) of 72% (36/50) and a negative predictive value (NPV) of 97% (561/581) (Table 1). Malaria microscopy was performed for 40% (290/731) of whom 7% (21/290) had positive results. Compared to PCR, microscopy had a sensitivity of 50% (18/36), a specificity of 99% (251/254), a PPV of 86% (18/21) and a NPV of 93% (251/269).

**Table 1:** Performance of RDT and microscopy compared to PCR among patients hospitalized with fever in Zanzibar (total n = 820)

	RDT (n = 631)	Microscopy (n = 290)
	Percentage (n/total)	Percentage (n/total)
Sensitivity	64% (36/56)	50% (18/36)
Specificity	98% (561/575)	99% (251/254)
Positive predictive value	72% (36/50)	86% (18/21)
Negative predictive value	97% (561/581)	93% (251/269)

Numbers given for patients investigated with PCR and each test. Discrepancies are due to missing values

RDT, rapid diagnostic test; PCR, polymerase chain reaction

**Fig. 3:**
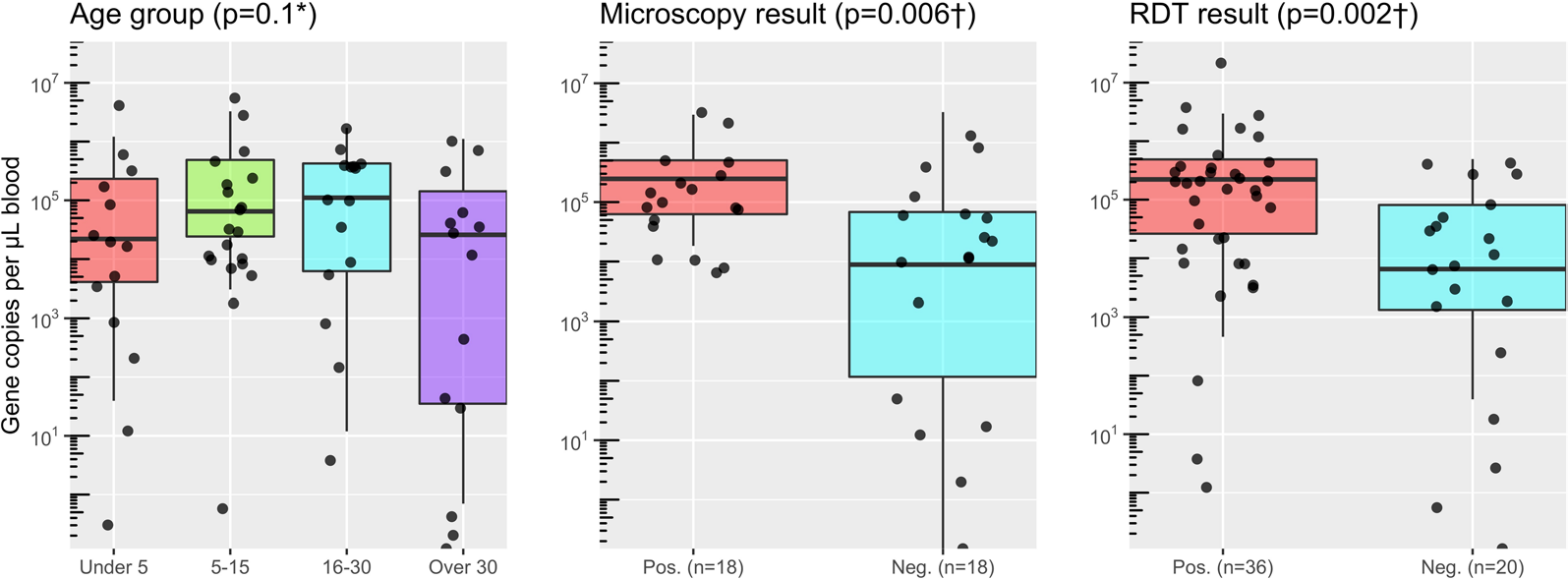
Malaria parasitaemia by age group (years) and result of microscopy and malaria rapid diagnostic test (RDT). Quantitation of parasitaemia by real-time PCR by diagnostic modalities expressed as log-transformed values of copies per µL blood. Unit of measurement for parasitaemia by PCR is described in the section PCR methods. Dots represent individual observations. Number tested in brackets. * Kruskal Wallis test † Wilcoxon rank sum test

Malaria patients with false negative microscopy results had significantly lower parasitaemia (median 9 × 10^3^ copies/µL, IQR 8 × 10^2^ – 7 × 10^4^) than the true positive ones (microscopy and PCR positive, median 2 × 10^5^ copies/µL, IQR 6 × 10^4^ – 5 × 10^5^, p = 0.006). Similarly, patients with false negative RDT had significantly lower parasitaemia (median 7 × 10^3^ copies/µL, IQR 2 × 10^3^ – 8 × 10^4^) than the true positive ones (median 2 × 10^5^ copies/µL, IQR 3 × 10^4^ – 5 × 10^5^, p = 0.002 (Fig. 3).

**Fig. 4:**
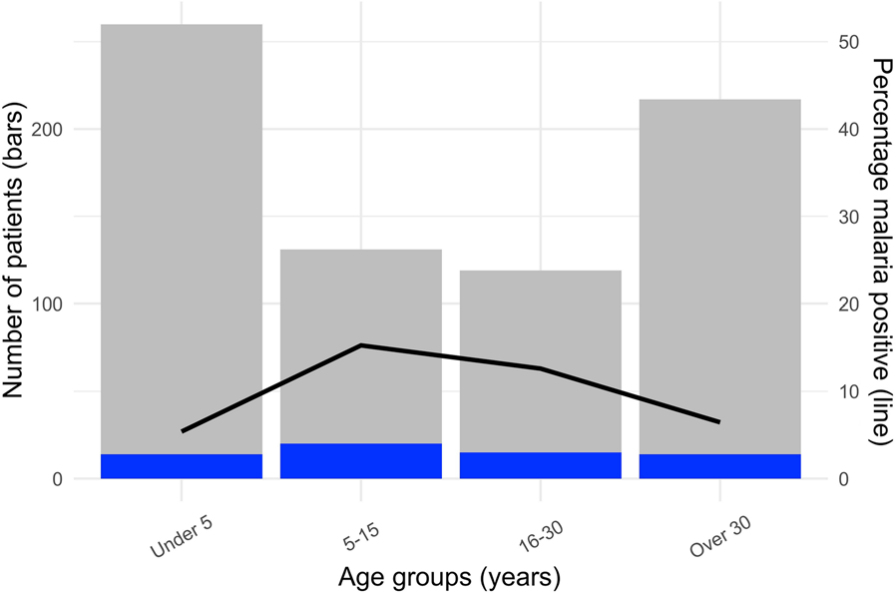
Malaria cases by age groups. Number of malaria patients (blue bars) among febrile patients (grey bars) and percentage positive (line) in different age groups

Malaria prevalence was 10% (41/421) in males and 7% (22/310) in females, however, this difference was not statistically significant (OR 1.4, CI 0.8–2.4, p= 0.3). The prevalence of malaria among children under 5 years old (5%, 14/260) was significantly lower compared to children aged 5 to 15 years (15%, 20/131, p = 0.001) and young adults aged 16 to 30 years (13%, 15/119, p = 0.02), but not different from that in people over 30 years old (6%, 14/217, p = 0.7, Fig. 4). However, the level of parasitaemia was not significantly different across the age groups (Table 2).

**Table 2:** Comparison of malaria prevalence and parasitaemia by age groups

Age group	Positive	OR (CI)p*	Parasitaemia in copies/µL median (IQR)	p^†^
Under 5	5% (14/260)	ref.	22 (4-236)	ref.
5-15 years	15% (20/131)	1.10 (1.04-1.17) **0.001**	65 (25-488)	0.30
16-30 years	13% (15/119)	1.07 (1.01-1.14) **0.020**	111 (6-427)	0.41
Over 30	6% (14/217)	1.01 (0.96-1.06) 0.678	28 (0.05-170)	0.65

* Logistic regression (glm in R)

^†^Kruskal–Wallis test and pairwise Wilcoxon rank sum test for multiple comparisons

**Fig. 5:**
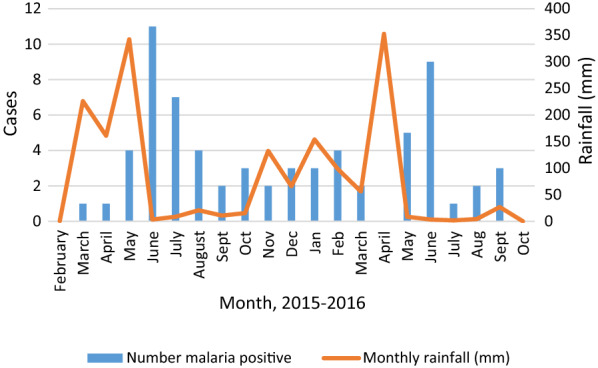
Monthly number of malaria cases and monthly rainfall from February 2015 to October 2016. Rainfall data for Dar es Salaam from the Tanzanian Meteorological Agency (TMA) [35, 36]. Study start March 17, 2015, study end October 4, 2016

Travel history was recorded for 33% (243/731). Malaria patients were more likely to have travelled to mainland Tanzania within the past six months (47%, 9/19), than those testing negative (6%, 13/224, OR 32, CI 5–42, p < 0.0001). Ten of the 19 malaria patients with known travel history had not visited the mainland, four of these had negative RDT. However, all ten cases of presumed autochthonous malaria were positive on PCR with a median parasitaemia of 5.0 × 10^4^ copies/µL blood (IQR 1 × 10^2^ – 2 × 10^5^). Monthly variations in malaria prevalence are shown in Fig. 5. An increase of malaria cases was observed at the end and shortly after the rainy season.

## Discussion

A malaria prevalence of 9% was identified by PCR in this study on patients hospitalized for acute febrile illness in Zanzibar in 2015–2016. This is higher than in a survey in 2009 to 2010 at three hospitals in Pemba, the second largest island of the Zanzibar archipelago, where only 1% of febrile patients were positive for malaria with RDT and/or microscopy [17].

There are no previous PCR-based malaria prevalence studies in hospitalized patients in Zanzibar. Previous community-based studies in Zanzibar reported a prevalence below 3% up to 2015, including PCR-based studies [18–20]. In 2015, a PCR-based study documented a 2% malaria prevalence in out-patients from rural areas of the two main islands of Zanzibar [5].

In line with that publication [5], the present study found a lower malaria prevalence in children under 5 years compared to school aged children and young adults (Fig. 4; Table 2). The relatively lower malaria prevalence in children < 5 years may be related to a higher probability of using insecticide-treated nets [5]. The higher prevalence in school age children and younger adults may reflect higher exposure to mosquitos during hours of transmission, but could also be related to a relatively lower immunity in this group, compared to the potentially semi-immune older population who were exposed to malaria prior to implementation of the comprehensive malaria control programme.

Malaria was strongly associated with travel to mainland Tanzania within the past six months. This is also shown in recent molecular studies substantiating malaria import from the mainland [21, 22]. However, ten patients had no travel history, confirming autochthonous malaria transmission inside the Zanzibar archipelago in the years 2015 and 2016. Four of these had negative RDT and would have been missed by routine diagnostics. In a survey of out-patients from rural areas of the two main islands of Zanzibar in the period from 2003 to 2015, findings imply ongoing autochthonous transmission [5]. Considering the presence of the effective malaria vector *Anopheles gambiae*, and increasing resistance of vectors to pyrethroid [23], re-introduced malaria can spread quickly in the population.

The finding of rising malaria prevalence shortly after rainy periods is in line with the study from Zanzibar [5] and numerous other studies.

The present study indicates that malaria had resurged as an important cause of febrile illness in Zanzibar by 2015. While fifteen years of comprehensive malaria control greatly reduced malaria incidence in the archipelago, it may also have rendered school age children and young adults with less immunity and increased the susceptibility to malaria. The finding of higher malaria prevalence in these age groups underlines the risk of severe malaria in a non-immune population.

The RDT showed a slightly poorer performance compared to PCR than reported previously from Zanzibar [18]. The lower sensitivity of RDTs in the present study (64%) compared to 77% in a study of Shakely et al. [18] may be explained by an inferior performance of the RDTs. In a WHO evaluation, the sensitivity for detecting 200 *P. falciparum* parasites per µL was scored 85% and 90% for the tests used in the present study (RDT First Response Malaria Ag. pLDH/HRP2 Combo Card Test and CareStartTM Malaria HRP2/pLDH (Pf/PAN) Combo Test) [24]. In comparison, the Paracheck Pf Test (Orchid Biomedical Systems, Goa, India) used in the study by Shakely et al. [18] had a sensitivity of 96% [24]. However, a limitation of studies comparing the performance of RDTs, is that the different PCR assays used as gold standards may have varying limits of detection. Thus, the very low detection limit of the PCR assay used in the current study [11] could, at least partly, explain the apparent lower sensitivity of the RDT in this study. The false-positivity rate in the WHO-evaluation of the three tests was 0.0%, 0.4% and 1.3%, respectively [24]. In the present study 36% of malaria cases were missed by RDT and 50% by microscopy compared to PCR. Since PCR has higher sensitivity in low level parasitaemia, it is possible that some of the discrepancy between PCR and RDT/microscopy could be due to coincidental non-significant low-level parasitaemia in semi-immune individuals suffering from febrile illness of other causes. Indeed, patients positive only by PCR had significantly lower parasitaemia than those who also had positive RDT and/or microscopy (Fig. 1). The limitation of RDT in low level parasitaemia is in line with a study reporting 34% sensitivity of RDT compared to PCR in reactive case detection programs in Zanzibar [22].

The superior sensitivity of PCR compared to microscopy is well known [9], and may, apart from inherent methodological issues, be due to suboptimal staining of blood slides, malfunctioning microscopes and deficient training of the laboratory technician [25]. In the present study, sensitivity of microscopy is still substantially higher than in several other surveys [26–29]. Our findings are in line with a review comparing PCR and microscopy for malaria diagnosis in endemic areas, which found that PCR identified on average twice the number of malaria infections compared to microscopy [30]. While PCR is highly sensitive, the level of parasitaemia detected by RDT and microscopy corresponds well with clinically relevant malaria [31].

With its high sensitivity, PCR may be useful in malaria surveillance, including reactive case detection in elimination programs as shown in recent publications from Zanzibar [22, 32]. In a study from 2015, PCR was positive in 2% of asymptomatic individuals in Zanzibar [33]. For clinical diagnosis of acute undifferentiated febrile illness, limitations of PCR are a longer turn-around time, higher cost and higher technical requirements than RDTs, as well as the potential for detecting non-significant low level malaria parasitaemia, or DNA remains of non-viable parasites weeks after parasite clearance [34].

## Conclusions

The study emphasizes the importance of malaria as a cause of febrile illness in patients admitted to hospital in Zanzibar, and confirms autochthonous malaria-transmission in Zanzibar in the years 2015 and 2016. The higher malaria prevalence in school age children and young adults could partly be related to waning immunity during the last decades of strict malaria control, in addition to factors such as behaviour and mosquito exposure. The study shows that currently used routine diagnostics may miss up to one-third of malaria positive patients in Zanzibar. Low sensitivities of routine diagnostic tests were related to poor test performance in patients with low parasitaemia.

## Data Availability

The datasets used during the current study are available from the corresponding author on reasonable request.

## Abbreviations

IQR: Interquartile range
MMH: Mnazi Mmoja Hospital
NPV: Negative predictive value
PCR: Polymerase chain reaction
PPV: Positive predictive value
RDT: Rapid diagnostic test
